# When Thyrotropin Meets Growth Hormone: The Clinical Challenges of a Mixed GH/TSH-Secreting PIT1-Lineage Pituitary Neuroendocrine Tumour

**DOI:** 10.3390/jcm15218533

**Published:** 2026-11-02

**Authors:** Alice Monsanto, Bárbara Jesus, Adriana Duarte, Mariana Lavrador, Mara Ventura, Daniela Guelho, Leonor Gomes

**Affiliations:** Department of Endocrinology, Diabetes and Metabolism, Unidade Local de Saúde de Coimbra, 3004-561 Coimbra, Portugal; barbarajesus1709@gmail.com (B.J.); adrianamrduarte@gmail.com (A.D.); mariana.lavrador@hotmail.com (M.L.); maracventura@gmail.com (M.V.); daniela_guelho@hotmail.com (D.G.); leonorgomes@hotmail.com (L.G.)

**Keywords:** acromegaly, growth hormone, thyrotropin, pituitary neuroendocrine tumour, PIT1, plurihormonal PitNET

## Abstract

**Background**: Acromegaly is most commonly caused by a growth hormone (GH)-secreting pituitary neuroendocrine tumour (PitNET). Plurihormonal PIT1-lineage PitNETs are uncommon, and combined GH and thyroid-stimulating hormone (TSH) secretion poses distinctive diagnostic and therapeutic challenges. We report a mixed GH/TSH-secreting PIT1-lineage PitNET in which the biochemical response to preoperative lanreotide supported the clinical suspicion of a functionally relevant thyrotroph component before targeted pathological reassessment. **Case Presentation**: A 39-year-old woman presented with a two-year history of progressive acral enlargement, headaches, hyperhidrosis, snoring, and voice deepening. Biochemical evaluation showed IGF-1 1128 ng/mL (reference range 93.1–281), GH 61 µg/L, TSH 1.5 µIU/mL (0.35–4.94), FT4 1.66 ng/dL (0.7–1.5), and FT3 7.0 pg/mL (1.8–4.2). Pituitary MRI demonstrated a 15 × 14 × 10.5 mm macroadenoma. Lanreotide 120 mg every four weeks reduced IGF-1 to 846 ng/mL and GH to 14 µg/L while normalizing FT4 (1.04 ng/dL) and FT3 (3.9 pg/mL), reinforcing the suspicion of mixed GH/TSH secretion. Transsphenoidal surgery was performed in October 2024. The tumour was initially classified as a densely granulated somatotroph PitNET; prompted by the preoperative biochemical phenotype, additional β-TSH immunostaining demonstrated positivity in numerous tumour cells, leading to reclassification as a mature plurihormonal PIT1-lineage PitNET (Ki-67 approximately 1%). Postoperative biochemical remission was not achieved, and MRI demonstrated a 7 mm residual lesion. Lanreotide was resumed, with the sustained control of the thyrotroph component but persistent GH/IGF-1 excess. Because residual disease persisted with slight cavernous sinus extension, Gamma Knife radiosurgery was performed in July 2026. **Conclusions**: Mixed GH/TSH-secreting PIT1-lineage PitNETs may be overlooked when TSH is inappropriately normal rather than frankly elevated. This case illustrates the value of integrating biochemical findings, treatment response, and targeted histopathological reassessment. Persistent disease despite surgery and somatostatin receptor ligand therapy further highlights the frequent need for multimodal, long-term management.

## 1. Introduction

Acromegaly is a rare chronic endocrine disorder caused in more than 95% of cases by a growth hormone (GH)-secreting pituitary neuroendocrine tumour (PitNET). Persistent GH hypersecretion results in elevated insulin-like growth factor 1 (IGF-1) concentrations and is associated with substantial cardiovascular, metabolic, musculoskeletal and neoplastic morbidity, leading to increased mortality if inadequately treated. Early diagnosis and prompt biochemical control are therefore essential to improve long-term outcomes [1,2,3].

Recent advances in pituitary tumour classification have substantially improved the understanding of pituitary neoplasia. The 2022 World Health Organization (WHO) Classification recognizes lineage-specific transcription factors as the cornerstone of PitNET classification, with PIT1-lineage tumours comprising somatotroph, lactotroph, thyrotroph and plurihormonal neoplasms. This classification better reflects tumour biology and has highlighted the heterogeneity of PIT1-derived tumours, including those with mixed hormonal secretion [4,5,6,7,8,9,10].

Mixed GH/TSH-secreting PitNETs are an uncommon subtype of PIT1-lineage tumours and are represented mainly by individual case reports and small retrospective series [11,12,13,14,15,16,17,18,19]. Their diagnosis may be particularly difficult because central hyperthyroidism is often subtle, with serum thyroid-stimulating hormone (TSH) concentrations remaining detectable or within the reference range despite elevated free thyroid hormone levels. Importantly, the immunohistochemical expression of more than one pituitary hormone does not, by itself, establish clinically relevant multihormonal hypersecretion; pathological findings must therefore be interpreted together with the biochemical and clinical phenotype. Current European Thyroid Association guidelines recommend considering a TSH-secreting PitNET whenever free thyroxine (FT4) and/or free triiodothyronine (FT3) concentrations are elevated in the presence of a non-suppressed TSH, after the appropriate consideration of analytical interference and resistance to thyroid hormone β [7,20,21,22,23].

Available evidence regarding GH/TSH co-secreting PitNETs remains limited. Most published data derive from isolated case reports, whereas only a few contemporary series have specifically evaluated their clinicopathological characteristics and outcomes. These tumours appear to present more frequently as macroadenomas and exhibit greater invasiveness and lower rates of surgical remission than pure somatotroph adenomas, frequently requiring multimodal treatment [19,24,25,26,27,28].

First-generation somatostatin receptor ligands (SRLs) represent an established treatment for both acromegaly and TSH-secreting PitNETs because of the high expression of somatostatin receptor subtypes 2 and 5. Besides inducing biochemical control and tumour shrinkage, the response to SRLs may provide valuable diagnostic information in patients with suspected mixed GH/TSH secretion before definitive histopathological confirmation. However, this potential diagnostic role has received relatively little attention in the literature [2,7,20,29,30].

Objective: The objective of this study was to describe the diagnostic and therapeutic course of a patient with a PIT1-lineage tumour showing GH and β-TSH expression, presenting clinically with acromegaly and biochemically with inappropriate TSH secretion, and to illustrate the value and limitations of clinicopathological correlation in establishing a functionally relevant mixed secretory phenotype.

## 2. Case Presentation

A 39-year-old woman was referred to our Endocrinology Department after acromegalic features were recognized during an emergency department assessment for abdominal pain and vomiting in October 2023. Retrospectively, she reported approximately two years of progressive acral enlargement, including an increase in shoe size from 38 to 40, together with voice deepening, hyperhidrosis, oily skin, snoring, progressive coarsening of facial features, and long-standing headaches. Her medical history was notable for insulin-treated gestational diabetes during a spontaneous twin pregnancy approximately three years before diagnosis; she had not undergone assisted reproductive treatment. She was receiving combined hormonal contraception when the acromegaly-related symptoms developed.

Physical examination revealed frontal bossing, broadening of the nose, prominent nasolabial folds, thickened lips, mandibular prognathism, macroglossia, interdental spacing and malocclusion, and enlargement of the hands and feet. The thyroid was diffusely enlarged, with a palpable right-sided nodular component. At the first assessment at our institution, weight was 67.8 kg, height 160 cm (BMI 26.5 kg/m^2^), blood pressure 146/80 mmHg, and heart rate 100 beats/min. HbA1c had been 5.8% in October 2023. In February 2024, fasting plasma glucose was 136 mg/dL, HbA1c 5.2%, and total serum calcium 9.2 mg/dL. An electrocardiogram showed sinus rhythm at 92 beats/min, with a corrected QT interval (QTc) of 437 ms. Ophthalmological assessment before surgery showed preserved visual acuity (10/10 bilaterally), no relative afferent pupillary defect, normal colour vision and fundoscopic examination, and no evidence of visual pathway involvement.

Biochemical evaluation in February 2024 showed IGF-1 575 ng/mL (reference range 63–223), with a concomitant GH concentration of 25 µg/L, TSH 0.8 µIU/mL, and FT4 1.81 ng/dL (reference range 0.7–1.5). The characteristic clinical phenotype together with clearly elevated age-adjusted IGF-1 supported the diagnosis of acromegaly; an oral glucose tolerance test for GH suppression was not performed. In May 2024, IGF-1 was 1128 ng/mL (reference range 93.1–281; 4.01 × upper limit of normal [ULN]), with GH 61 µg/L. In parallel, TSH was inappropriately normal at 1.5 µIU/mL (0.35–4.94) with elevated FT4 1.66 ng/dL (0.7–1.5) and FT3 7.0 pg/mL (1.8–4.2). Thyroid autoantibodies were negative, including TRAb (1.1; reference <2 in March 2024). Prolactin was 13.6 ng/mL, ACTH 7.4 pg/mL, and morning cortisol 6.5 µg/dL. The hypothalamic–pituitary–adrenal axis had previously been assessed with a standard 250 µg intramuscular Synacthen test, with cortisol increasing from 10 to 33 µg/dL at 60 min.

Pituitary magnetic resonance imaging in February 2024 revealed a well-defined left parasagittal sellar macroadenoma measuring 15 × 14 × 10.5 mm. The lesion displaced the pituitary stalk to the right and elevated the superior glandular contour without optic chiasm compression. There was loss of the cleavage plane with the medial margin of the left cavernous sinus, although definite cavernous sinus invasion was not identified radiologically (Figure 1A,B).

Because thyroid hormone concentrations were elevated with a non-suppressed TSH, inappropriate pituitary TSH secretion was suspected. Thyroid ultrasound showed an enlarged heterogeneous multinodular gland, and the fine-needle aspiration of a dominant right-lobe nodule measuring 2.1 × 1.0 × 2.7 cm showed a benign colloid/hyperplastic adenomatous nodule (Bethesda II).

The case was discussed by the multidisciplinary pituitary team, and transsphenoidal surgery remained the planned definitive first-line treatment. While awaiting surgery, lanreotide was initiated at 120 mg every four weeks as bridging therapy to reduce the marked GH/IGF-1 excess and improve symptoms; one administration was reduced to 90 mg because of temporary drug unavailability. By September 2024, after approximately four months of treatment, IGF-1 had decreased to 846 ng/mL (reference range 93.1–281; 3.01 × ULN), with a concomitant GH concentration of 14 µg/L. In parallel, TSH decreased to 0.76 µIU/mL, and FT4 normalized to 1.04 ng/dL and FT3 to 3.9 pg/mL. The synchronous improvement in the somatotroph and thyrotroph biochemical profiles provided supportive, although not definitive, evidence of a functionally relevant mixed secretory phenotype.

Transsphenoidal surgery was performed on 10 October 2024. Intraoperatively, subtotal tumour removal was achieved, with a probable residual component in the left cavernous sinus. This finding was concordant with the preoperative loss of the cleavage plane at the medial cavernous sinus despite the absence of definite radiological invasion. Three days after surgery, TSH was 0.11 µIU/mL, and IGF-1 was 401 ng/mL (reference range 93.1–281; 1.43 × ULN); FT4 was 0.85 ng/dL on postoperative day 4. These early postoperative measurements were not used to define remission. By November 2024, persistent acromegaly was evident, with IGF-1 789 ng/mL (reference range 93.1–281; 2.81 × ULN) and a concomitant GH concentration of 9.4 µg/L, while TSH was 0.43 µIU/mL and FT4 1.28 ng/dL.

Initial histopathological examination showed fragments of an adenohypophyseal tumour with a diffuse growth pattern, composed of round-to-polygonal cells with variably eosinophilic, clear, and occasionally acidophilic cytoplasm. No mitotic figures were identified. Tumour cells showed nuclear PIT1 positivity and diffuse cytoplasmic expression of GH and CAM5.2, with perinuclear CAM5.2 staining; TPIT and GATA3 were negative, and the Ki-67 proliferative index was approximately 1%. The lesion was initially classified as a densely granulated somatotroph PitNET. Given the preoperative biochemical suspicion of inappropriate TSH secretion, a targeted pathological review was requested. Additional β-TSH immunostaining showed cytoplasmic positivity in numerous tumour cells, leading to reclassification as a mature plurihormonal PIT1-lineage PitNET. A broader immunohistochemical panel for all pituitary hormones was not available.

The postoperative course was complicated by suspected meningitis requiring readmission in November 2024 and six weeks of intravenous antimicrobial therapy. At approximately four months after surgery, biochemical activity persisted: on 25 February 2025, IGF-1 was 794 ng/mL (2.83 × ULN), GH 11 µg/L, TSH 0.66 µIU/mL, FT4 1.71 ng/dL, and FT3 4.5 pg/mL. Repeat thyroid testing the following day again showed central hyperthyroidism (TSH 0.97 µIU/mL, FT4 1.62 ng/dL, FT3 5.7 pg/mL), with negative TSH receptor antibodies. A repeat Synacthen test showed an adequate cortisol response to 26 µg/dL at 60 min. Postoperative MRI demonstrated partial tumour resection with a residual lesion measuring 7 mm in the greatest longitudinal diameter (Figure 1C). Lanreotide 120 mg every four weeks was therefore resumed.

Under continued lanreotide therapy, the thyroid biochemical abnormalities became controlled, whereas acromegaly remained active. In June 2025, IGF-1 was 478 ng/mL (reference range 93.1–281; 1.70 × ULN), with GH 9.0 µg/L, TSH 0.48 µIU/mL and FT4 1.41 ng/dL. In November 2025, IGF-1 was 527 ng/mL (1.88 × ULN), with GH 6.2 µg/L, while TSH, FT4, and FT3 were 0.63 µIU/mL, 1.13 ng/dL, and 4.2 pg/mL, respectively. In February 2026, IGF-1 remained elevated at 539 ng/mL (1.92 × ULN), with GH 11 µg/L, whereas TSH 0.75 µIU/mL, FT4 1.23 ng/dL, and FT3 3.9 pg/mL were within the reference range. In April 2026, IGF-1 was 550 ng/mL (1.96 × ULN), with GH 7.3 µg/L, TSH 0.48 µIU/mL and FT4 1.31 ng/dL. The longitudinal biochemical and therapeutic course is summarized in Table 1, showing the sustained normalization of the thyroid axis after lanreotide reintroduction despite persistent IGF-1 elevation.

Follow-up MRI in February 2026 showed a morphologically stable residual lesion involving the left pituitary gland, with apparent slight extension towards the cavernous sinus but without the compromise of the left internal carotid artery. Given persistent biochemical and radiological disease despite surgery and first-generation somatostatin receptor ligand therapy, the case was again reviewed by the multidisciplinary team. Gamma Knife stereotactic radiosurgery was performed on 2 July 2026, with continued biochemical and imaging surveillance planned (Figure 1D).

Preliminary findings from this case, including the initial clinical presentation and biochemical profile, the response to preoperative lanreotide, the histopathological findings, and the early postoperative assessment of residual disease, were presented as poster PD19, entitled “When Thyrotropin Meets Growth Hormone: Clinical Challenges of a mixed GH/TSH adenoma”, at the 77th Annual Meeting of the Portuguese Society of Endocrinology, Diabetes and Metabolism (SPEDM), held in Coimbra, Portugal, on 30 January 2026. The present report additionally describes the subsequent biochemical and radiological follow-up and Gamma Knife radiosurgery performed in July 2026.

## 3. Discussion

Mixed GH/TSH-secreting PIT1-lineage PitNETs are rare and clinically challenging tumours in which manifestations of GH excess may dominate the presentation while biochemical evidence of inappropriate TSH secretion remains subtle. In this case, the combination of overt acromegaly, elevated FT4 and FT3 with a non-suppressed TSH, synchronous improvement in both biochemical profiles during lanreotide therapy, and subsequent β-TSH immunoreactivity supported a clinically relevant mixed phenotype. However, because analytical interference, α-subunit measurement and THRB testing were not formally assessed, these findings support rather than unequivocally prove biologically autonomous TSH hypersecretion.

The recognition of central hyperthyroidism depends on interpreting TSH in relation to circulating thyroid hormone concentrations rather than relying on whether TSH lies within the laboratory reference interval. Elevated FT4 and/or FT3 with a detectable or non-suppressed TSH should raise the suspicion of inappropriate TSH secretion and prompt consideration of analytical interference, resistance to thyroid hormone β, and a TSH-secreting PitNET [20,22,23]. In the present patient, TSH was repeatedly non-suppressed despite elevated FT4 and FT3, and the clinical phenotype was dominated by acromegaly rather than overt thyrotoxicosis. The concomitant multinodular goitre could have distracted from a central cause; however, benign cytology, negative thyroid autoantibodies, the reproducible response of thyroid hormones to lanreotide, and ultimately β-TSH expression by tumour cells strongly supported pituitary TSH secretion. Analytical interference and THRB testing were not formally investigated and therefore cannot be stated to have been biochemically excluded.

The pathological sequence is another instructive feature of this case. The 2022 WHO classification emphasizes lineage-specific transcription factors in PitNET classification, with somatotroph, lactotroph, thyrotroph, and mature plurihormonal tumours belonging to the PIT1 lineage [4,5,6,9]. Initial histology showed a PIT1-positive, diffusely GH- and CAM5.2-positive tumour with a low Ki-67 index and no mitotic figures, leading to classification as a densely granulated somatotroph PitNET. Because the biochemical phenotype suggested concomitant inappropriate TSH secretion, additional β-TSH staining was specifically requested and was positive in numerous tumour cells, resulting in reclassification as a mature plurihormonal PIT1-lineage PitNET. This distinction had clinical relevance beyond nomenclature: it prevented the abnormal thyroid profile from being attributed solely to primary thyroid disease in a patient with multinodular goitre, prompted targeted pathological reassessment, supported the monitoring of both secretory axes, and informed the interpretation of the differential response to treatment. Conversely, β-TSH immunoreactivity alone should not be equated with clinically significant TSH hypersecretion, underscoring the need for clinicopathological correlation.

Evidence specific to GH/TSH co-secreting PitNETs remains limited, with much of the literature consisting of case reports and small retrospective cohorts [11,12,13,14,15,16,17,18,19]. In the series by Yu et al., which included 21 patients with GH/TSH co-secreting tumours, all lesions were macroadenomas; compared with matched pure GH-secreting PitNETs, GH/TSH tumours had larger diameters, more frequent cavernous sinus invasion, lower rates of gross total resection, and lower long-term remission rates [19]. The more recent case–control study by Mo et al. likewise supports a distinct and therapeutically challenging phenotype of GH/TSH co-secretion [28]. These observations do not establish uniformly aggressive biological behaviour, but they suggest that the recognition of a mixed secretory phenotype may affect surgical expectations and reinforce the need for the prolonged surveillance of both GH/IGF-1 and thyroid function. Our patient’s course is concordant with this pattern: subtotal resection left a probable cavernous sinus remnant, biochemical disease persisted, and long-term management required continued medical therapy followed by stereotactic radiosurgery.

Somatostatin receptor ligands are particularly relevant in mixed GH/TSH tumours because both somatotroph and thyrotroph PitNETs commonly respond to first-generation agents. In thyrotroph PitNETs, somatostatin analogues can suppress inappropriate TSH secretion, restore euthyroidism, and may reduce tumour volume; they are used preoperatively and as adjunctive treatment when surgery is incomplete or unsuccessful [20,29,30]. In our patient, lanreotide was used as bridging therapy while awaiting surgery. After approximately four months, IGF-1 decreased from 1128 to 846 ng/mL, and FT4 and FT3 normalized; concomitant GH concentrations also decreased from 61 to 14 µg/L, although random GH values were not used as a standardized diagnostic or response endpoint. Yu et al. also reported the marked suppression of both GH and TSH during octreotide suppression testing in GH/TSH co-secreting tumours [19]. Therapeutic response cannot independently establish co-secretion, but the synchronous biochemical improvement provided functional support for the hypothesis of a single mixed secretory PitNET before histopathological reassessment.

The postoperative biochemical trajectory provides additional insight. IGF-1 fell to 401 ng/mL (1.43 × ULN) three days after surgery, but such an early measurement was not used to define remission. Subsequent measurements documented persistent disease, with IGF-1 789 ng/mL in November 2024 and 794 ng/mL in February 2025. The biochemical pattern of central hyperthyroidism also recurred by February 2025. MRI confirmed a 7 mm residual tumour (Figure 1C), prompting the reintroduction of lanreotide. Thereafter, thyroid function remained controlled, whereas IGF-1 remained persistently elevated at approximately 1.7–2.0 × ULN. This differential biochemical response is clinically noteworthy and could reflect intratumoral heterogeneity in somatostatin receptor expression or downstream signalling; however, receptor profiling was not available, and this mechanistic interpretation remains speculative.

The requirement for additional therapy should be interpreted in the context of residual tumour and secretory persistence rather than as evidence of an aggressive PitNET. In a meta-analysis of 536 patients with TSH-secreting pituitary tumours, pooled postoperative biochemical remission was approximately 70%, gross total resection was achieved in 54%, and cavernous sinus invasion was associated with a lower likelihood of complete resection [25]. Importantly, overall biochemical remission increased substantially with adjuvant medical treatment and/or radiotherapy, supporting a multimodal approach when surgery is not curative [25]. In our patient, follow-up MRI demonstrated persistent residual disease with slight extension towards the cavernous sinus, while GH/IGF-1 excess persisted despite first-generation somatostatin receptor ligand therapy. Multidisciplinary review therefore led to Gamma Knife radiosurgery in July 2026. Long-term biochemical and radiological surveillance remains necessary because the endocrine response to radiosurgery may be delayed.

In the present case, the recognition of the mixed GH/TSH phenotype was particularly relevant for diagnostic characterisation, the interpretation of the differential biochemical response to treatment, and longitudinal endocrine surveillance. Although previous studies have associated GH/TSH co-secreting PitNETs with greater invasiveness and lower remission rates, therapeutic decisions in our patient were primarily driven by the established indications for acromegaly management. Transsphenoidal surgery had already been planned, lanreotide was initiated as bridging therapy for marked GH/IGF-1 excess, and subsequent radiosurgery was indicated by residual tumour and persistent somatotroph hypersecretion. Thus, while the recognition of the mixed phenotype provided relevant prognostic context and supported the importance of the prolonged surveillance of both secretory axes, it did not independently determine the therapeutic strategy or demonstrably influence clinical outcome.

This report has limitations. It describes a single patient, and α-subunit, sex hormone-binding globulin, a formal assessment of analytical interference, THRB genetic testing, somatostatin receptor subtype profiling, and a complete immunohistochemical panel for all pituitary hormones were not available. An oral glucose tolerance test for GH suppression was not performed; therefore, random GH concentrations are reported only as descriptive longitudinal data and were not used as a standardized diagnostic or response criterion. Additional investigations such as thyroid scintigraphy or somatostatin receptor imaging were not performed in this real-world clinical setting. Accordingly, β-TSH immunoreactivity establishes TSH expression within a plurihormonal PIT1-lineage tumour, while the functional significance of the thyrotroph component is supported—but not unequivocally proven—by the repeated biochemical pattern of elevated free thyroid hormones with non-suppressed TSH and its reproducible response to lanreotide. Nevertheless, the case provides detailed longitudinal biochemical, radiological, therapeutic, and pathological data and illustrates how biochemical suspicion can prompt targeted histopathological reassessment and clinically relevant dual-axis follow-up.

## 4. Conclusions

Mixed GH/TSH-secreting PIT1-lineage PitNETs should be considered when acromegaly coexists with elevated free thyroid hormone concentrations and a non-suppressed or inappropriately normal TSH. This case demonstrates the importance of integrating biochemical findings, treatment response, and pathology. The recognition of the possible thyrotroph component prompted targeted β-TSH immunostaining and reclassification from a densely granulated somatotroph PitNET to a mature plurihormonal PIT1-lineage tumour while also supporting the longitudinal monitoring of both secretory axes. Because β-TSH immunoreactivity alone does not prove biologically autonomous TSH secretion, the functional diagnosis should remain grounded in the complete biochemical and clinical context. Persistent IGF-1 excess after surgery and long-term lanreotide, ultimately requiring Gamma Knife radiosurgery, further illustrates the potential need for individualized multimodal treatment and prolonged biochemical and radiological surveillance.

## Figures and Tables

**Figure 1:**
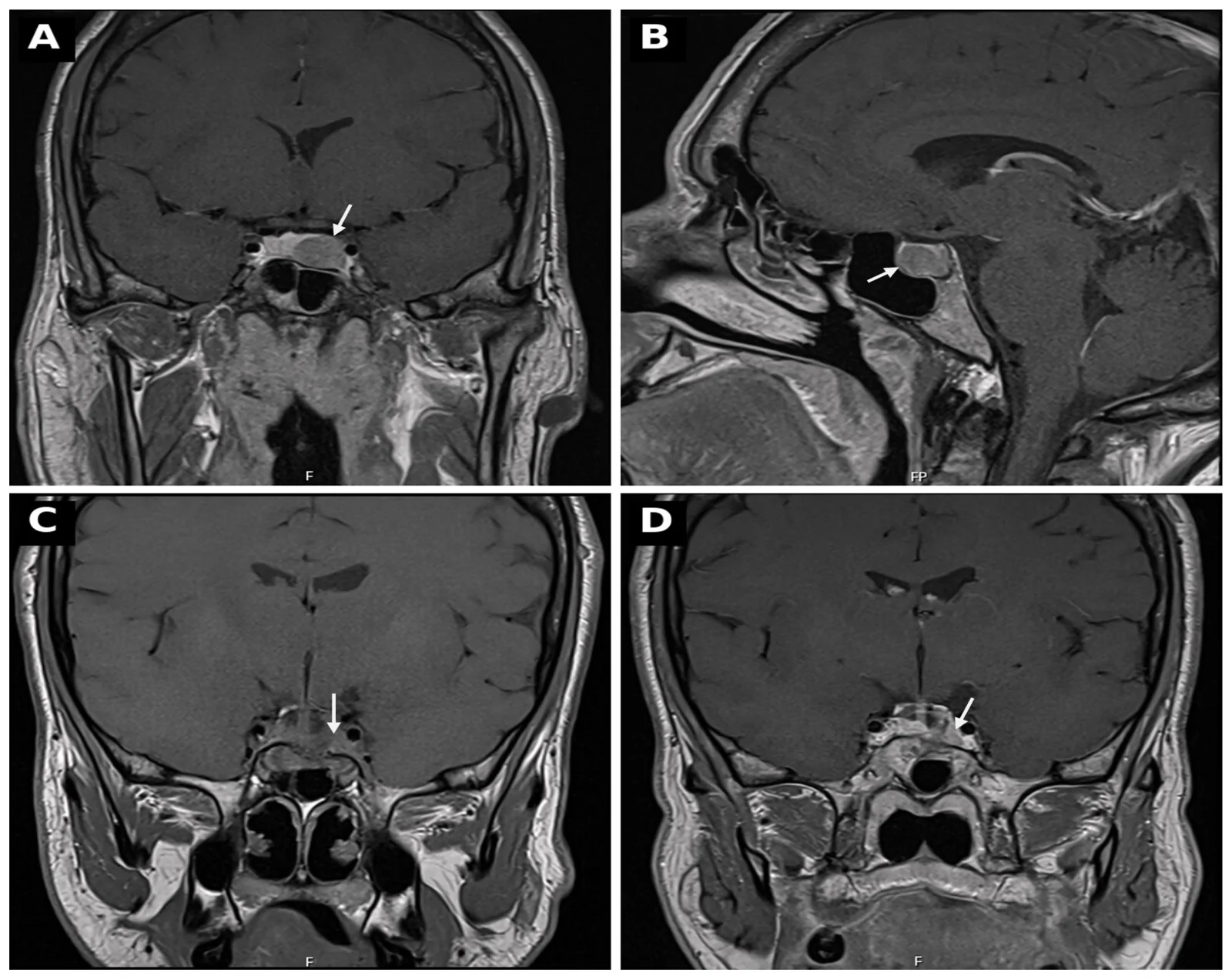
Evolution of pituitary magnetic resonance imaging findings. (**A**,**B**) Pre-treatment pituitary MRI (16 February 2024), showing sellar macroadenoma (arrows) in coronal and sagittal views. (**C**) Postoperative MRI (25 February 2025), showing residual pituitary lesion (arrow). (**D**) Follow-up MRI (27 February 2026), prior to Gamma Knife radiosurgery, showing persistence of residual lesion (arrow).

**Table 1:** Longitudinal biochemical evolution and treatment course.

Time Point	IGF-1, ng/mL (×ULN)	GH (µg/L)	TSH (µIU/mL)	FT4 (ng/dL)	FT3 (pg/mL)	Treatment/Imaging/Event
May 2024	1128 (4.01)	61	1.50	1.66	7.0	Biochemical diagnosis; MRI: 15 × 14 × 10.5 mm macroadenoma; before lanreotide
September 2024	846 (3.01)	14	0.76	1.04	3.9	Preoperative lanreotide while awaiting surgery
13–14 October 2024	401 (1.43)	—	0.11	0.85	—	Transsphenoidal surgery 10 October 2024; subtotal resection; early postoperative values
29 November 2024	789 (2.81)	9.4	0.43	1.28	—	Postoperative follow-up; persistent acromegaly
25 February 2025	794 (2.83)	11	0.66	1.71	4.5	MRI: 7 mm residual lesion; lanreotide restarted
23 June 2025	478 (1.70)	9.0	0.48	1.41	—	Lanreotide 120 mg/4 weeks
10 November 2025	527 (1.88)	6.2	0.63	1.13	4.2	Lanreotide 120 mg/4 weeks
3 February 2026	539 (1.92)	11	0.75	1.23	3.9	Lanreotide 120 mg/4 weeks; MRI February 2026: stable residual with slight cavernous sinus extension
27 April 2026	550 (1.96)	7.3	0.48	1.31	—	Lanreotide; before radiosurgery
2 July 2026	—	—	—	—	—	Gamma Knife radiosurgery

## Data Availability

The data presented in this study are available on request from the corresponding author. The data are not publicly available due to privacy and ethical restrictions.
